# Protective ventilation and outcomes of critically ill patients with COVID-19: a cohort study

**DOI:** 10.1186/s13613-021-00882-w

**Published:** 2021-06-07

**Authors:** Juliana C. Ferreira, Yeh-Li Ho, Bruno Adler Maccagnan Pinheiro Besen, Luiz Marcelo Sa Malbouisson, Leandro Utino Taniguchi, Pedro Vitale Mendes, Eduardo Leite Vieira Costa, Marcelo Park, Renato Daltro-Oliveira, Roberta M. L. Roepke, Joao M. Silva-Jr, Maria Jose Carvalho Carmona, Carlos R. R. Carvalho, EPICCoV Study Group, Adriana Hirota, Alberto Kendy Kanasiro, Alessandra Crescenzi, Amanda Coelho Fernandes, Anna Miethke-Morais, Arthur Petrillo Bellintani, Artur Ribeiro Canasiro, Bárbara Vieira Carneiro, Beatriz Keiko Zanbon, Bernardo Pinheiro Senna Nogueira Batista, Bianca Ruiz Nicolao, Bruno Adler Maccagnan Pinheiro Besen, Bruno Biselli, Bruno Rocha De Macedo, Caio Machado Gomes De Toledo, Carlos Roberto Ribeiro De Carvalho, Caroline Gomes Mol, Cassio Stipanich, Caue Gasparotto Bueno, Cibele Garzillo, Clarice Tanaka, Daniel Neves Forte, Daniel Joelsons, Daniele Robira, Eduardo Leite Vieira Costa, Elson Mendes Da Silva Júnior, Fabiane Aliotti Regalio, Gabriela Cardoso Segura, Giulia Sefrin Louro, Gustavo Brasil Marcelino, Yeh-Li Ho, Isabela Argollo Ferreira, Jeison Oliveira Gois, Joao Manoel Da Silva-Jr, Jose Otto Reusing Junior, Julia Fray Ribeiro, Juliana Carvalho Ferreira, Karine Vusberg Galleti, Katia Regina Silva, Larissa Padrao Isensee, Larissa Santos Oliveira, Leandro Utino Taniguchi, Leila Suemi Letaif, Lígia Trombetta Lima, Lucas Yongsoo Park, Lucas Chaves Netto, Luciana Cassimiro Nobrega, Luciana Bertocco Paiva Haddad, Ludhmila Abrahao Hajjar, Luiz Marcelo Sa Malbouisson, Manuela Cristina Adsuara Pandolfi, Marcelo Park, Maria José Carvalho Carmona, Maria Castilho Prandini H. Andrade, Mariana Moreira Santos, Matheus Pereira Bateloche, Mayra Akimi Suiama, Mayron Faria de Oliveira, Mayson Laercio Sousa, Michelle Louvaes Garcia, Natassja Huemer, Pedro Vitale Mendes, Paulo Ricardo Gessolo Lins, Pedro Gaspar Dos Santos, Pedro Ferreira Paiva Moreira, Renata Mello Guazzelli, Renato Batista Dos Reis, Renato Daltro-Oliveira, Roberta Muriel Longo Roepke, Rodolpho Augusto Moura Pedro, Rodrigo Kondo, Samia Zahi Rached, Sergio Roberto Silveira Da Fonseca, Thais Sousa Borges, Thalissa Ferreira, Vilson Cobello Junior, Vivian Vieira Tenório Sales, Willaby Serafim Cassa Ferreira

**Affiliations:** 1 Divisao de Pneumologia, Instituto Do Coracao, Hospital das Clinicas HCFMUSP, Faculdade de Medicina, Universidade de Sao Paulo, São Paulo, Brazil; 2 Intensive Care Unit, AC Camargo Cancer Center, São Paulo, Brazil; 3 Divisao de Molestias Infecciosas, Faculdade de Medicina, Hospital das Clinicas HCFMUSP, Universidade de Sao Paulo, Sao Paulo, SP Brazil; 4 Medical ICU, Disciplina de Emergências Clínicas, Departamento de Clínica Médica, Hospital das Clínicas HCFMUSP, Faculdade de Medicina, Universidade de São Paulo, São Paulo, SP Brazil; 5 Divisao de Anestesia, Hospital das Clinicas HCFMUSP, Faculdade de Medicina, Universidade de Sao Paulo, Sao Paulo, SP Brazil; 6 UTI Emergencias Cirurgicas E Trauma, Departamento de Cirurgia, Hospital das Clinicas HCFMUSP, Faculdade de Medicina, Universidade de Sao Paulo, Sao Paulo, SP Brazil

**Keywords:** Ventilation, artificial, Severe acute respiratory syndrome, SARS virus, Pneumonia, viral, COVID-19

## Abstract

**Background:**

Approximately 5% of COVID-19 patients develop respiratory failure and need ventilatory support, yet little is known about the impact of mechanical ventilation strategy in COVID-19. Our objective was to describe baseline characteristics, ventilatory parameters, and outcomes of critically ill patients in the largest referral center for COVID-19 in Sao Paulo, Brazil, during the first surge of the pandemic.

**Methods:**

This cohort included COVID-19 patients admitted to the intensive care units (ICUs) of an academic hospital with 94 ICU beds, a number expanded to 300 during the pandemic as part of a state preparedness plan. Data included demographics, advanced life support therapies, and ventilator parameters. The main outcome was 28-day survival. We used a multivariate Cox model to test the association between protective ventilation and survival, adjusting for PF ratio, pH, compliance, and PEEP.

**Results:**

We included 1503 patients from March 30 to June 30, 2020. The mean age was 60 ± 15 years, and 59% were male. During 28-day follow-up, 1180 (79%) patients needed invasive ventilation and 666 (44%) died. For the 984 patients who were receiving mechanical ventilation in the first 24 h of ICU stay, mean tidal volume was 6.5 ± 1.3 mL/kg of ideal body weight, plateau pressure was 24 ± 5 cmH_2_O, respiratory system compliance was 31.9 (24.4–40.9) mL/cmH_2_O, and 82% of patients were ventilated with protective ventilation. Noninvasive ventilation was used in 21% of patients, and prone, in 36%. Compliance was associated with survival and did not show a bimodal pattern that would support the presence of two phenotypes. In the multivariable model, protective ventilation (aHR 0.73 [95%CI 0.57–0.94]), adjusted for PF ratio, compliance, PEEP, and arterial pH, was independently associated with survival.

**Conclusions:**

During the peak of the epidemic in Sao Paulo, critically ill patients with COVID-19 often required mechanical ventilation and mortality was high. Our findings revealed an association between mechanical ventilation strategy and mortality, highlighting the importance of protective ventilation for patients with COVID-19.

**Supplementary Information:**

The online version contains supplementary material available at 10.1186/s13613-021-00882-w.

## Background

The pandemic of coronavirus disease (COVID-19) that arose in China in December 2019 has spread across the globe, causing more than 166 million cases on all continents as of May 2021 [1]. Brazil ranks second in number of deaths, and the city of São Paulo was the first and more severely affected city in the country, with over 29,000 deaths by the end of May 2021 [2].

Epidemiological studies reporting the outcomes of COVID-19 patients in China, Europe, and the United States showed a high mortality among critical patients [3–11] particularly for those who required invasive mechanical ventilation. More recently, reports described respiratory mechanics and ventilatory parameters applied to COVID-19 patients [12–24]. Findings of relatively normal respiratory compliance raised the question of whether COVID-19-associated Acute Respiratory Distress Syndrome (ARDS) is different from ARDS due to other causes, and led to the proposition of two distinct phenotypes [14–17]. However, the questions of whether respiratory mechanics and ventilatory parameters are associated with clinical outcomes and whether ventilatory strategies recommended for ARDS apply for COVID-19 are still a subject of debate [16, 25–27]. As the epidemic continues to take the lives of thousands of people every day around the globe, there is a need for data on ventilatory management of COVID-19 that can inform clinical decision at the bedside. Our objective was to describe baseline characteristics and ventilatory parameters, and to estimate the association of protective ventilation with outcomes of patients admitted to the ICUs of the largest public hospital in Sao Paulo, during the first surge of the pandemic of COVID-19 in Brazil.

## Methods

### Study design and location

This is a cohort study conducted at Hospital das Clínicas from University of Sao Paulo Medical School, the largest academic hospital in Brazil and primary referral center for critically ill patients with COVID-19 during the first surge of the pandemic. The study protocol was published elsewhere [28]. In brief, the largest building of an academic hospital complex with 94 ICU beds was dedicated to COVID-19. Operating rooms and hospital wards were converted into surge ICUs [29], resulting in 20 ICUs with 300 ICU beds.

The study was approved by the Research Ethics Committee of Hospital das Clínicas da Universidade de São Paulo and registered in a public registry (clinicaltrials.gov, NCT04378582). Informed consent was waived due to the observational nature of the study.

### Study population

We included all consecutive patients admitted to the ICU from March 30 to June 30, 2020. Inclusion criteria were cases of suspected or confirmed COVID-19 and age older than 14 years and exclusion criterion was ICU stay shorter than 24 h. Patients were included in the study only on their first ICU admission, and classified as confirmed COVID-19, highly suspected COVID-19, and ruled-out COVID-19 (more details in Additional file 1).

### Patient care

Since this was an observational study, patient care was not part of study procedures, but the hospital developed institutional protocols specifically for COVID-19 patients, including the use of personal protective equipment, ventilatory management, thrombosis prophylaxis, and sedation (more details in Additional file 1).

### Outcomes

The main outcome was survival at 28 days. Secondary outcomes included duration of mechanical ventilation, need for vasopressors or renal replacement therapy, and hospital survival at 60 days. We opted for a survival analysis instead of cumulative mortality, because COVID-19 is an acute disease, with a convalescence phase that needs time to occur, typically 2–3 weeks. If any intervention—such as protective ventilation—contributes to maintain critical patients alive long enough for the lung inflammation to subside, and the immune system to respond, extending survival can be advantageous from a patient´s perspective.

### Data collection

Data were collected prospectively from study approval (May 6) to July 28, 2020, and retrospectively from March 30 to May 5, 2020. We reviewed electronic medical records, laboratory results, and collected data at the bedside during morning rounds. Study data were collected and managed using a secure, web-based platform (REDCap—Research Electronic Data Capture) [30]. Data included demographic information, symptoms, comorbidities, Simplified Acute Physiology Score (SAPS3) [31], Sepsis-related Organ Failure Assessment (SOFA) [32], and laboratory tests at admission. Ventilatory parameters were collected on day 1, and included tidal volume, respiratory rate, inspired fraction of oxygen (FIO_2_), positive end-expiratory pressure (PEEP), and plateau pressure. Driving pressure was calculated as plateau pressure minus total PEEP. Respiratory system compliance was obtained by dividing tidal volume in mLs by the driving pressure. We also calculated compliance normalized by ideal body weight by dividing tidal volume, in mL/kg of ideal body weight by the driving pressure (see Additional file 1). Protective ventilation was defined as ventilation with tidal volume < 8 ml/Kg and plateau pressure < 30 cmH_2_O. Patients were followed for 60 days.

The results are reported in accordance to recommend the Strengthening The Reporting of Observational Studies in Epidemiology (STROBE) guidelines [33].

### Statistical analysis plan

A sample size of 300 patients was initially anticipated. However, as the epidemic in Sao Paulo grew fast, the hospital opened new ICU beds, and given that the study was observational and posed no risks for participants, we collected data for all patients with COVID-19 admitted to the ICUs during the study period.

Categorical variables are expressed as count and percentage, and continuous variables, as mean and standard deviation, or median and interquartile range (IQR) as appropriate.

We built Kaplan–Meier curves to estimate survival at 28 days. We performed survival analysis using the Cox proportional hazard model. Survival at 28 and 60 days was defined as the time interval between ICU admission and patient death from any cause or hospital discharge. Patients discharged home or transferred to another hospital were considered alive at the end of follow-up. Those still in hospital after July 28th, 2020 had their data censored.

We tested the association of protective mechanical ventilation and other relevant ventilatory variables with survival for patients under mechanical ventilation using Cox proportional hazard models. The multivariable model was based on a conceptual causal diagram including relevant covariates (Additional file 1: Figure S1), and tested the association between protective ventilation, adjusting for PF ratio, pH, compliance, and PEEP.

Associations between baseline characteristics and survival at 28 days resulted in multiple comparisons, which could lead to type I error, and therefore, we focused our main analysis on the associations between relevant ventilatory variables and survival. All other associations were considered exploratory and tested in additional multivariate models, as shown in Additional file 1. Sensitivity analyses were performed adding base excess and use of vasoactive drugs at admission to the multivariable Cox model and excluding suspected cases. All hypothesis tests are two-tailed with a significance level of 0.05 and performed using the R software (R Core Team, 2016, Vienna, Austria).

## Results

**Fig. 1:**
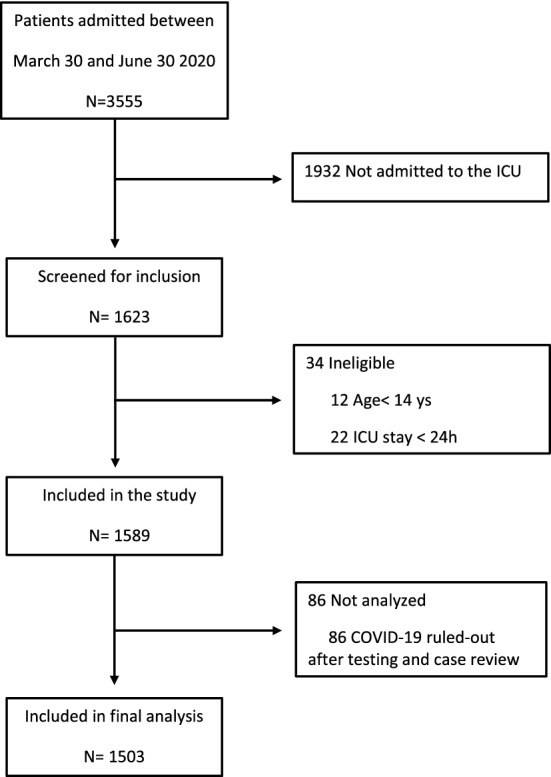
Study participant flow. Flow of potentially eligible participants in the study, and final numbers included and analyzed

Of 3555 consecutive patients with COVID-19 who were admitted to the hospital between March 30 and June 30, 2020, 1932 were excluded, because they were not admitted to the ICU, and 12 patients were younger than 14 years old and 22 stayed in the ICU for less than 24 h. Thus, 1589 patients were included in the study (Fig. 1). Of these, 86 had COVID-19 ruled out during follow-up, resulting in 1503 patients in the final analysis. Figure S2 (Additional file 1) shows that patients were transferred to our hospital from all regions of the metropolitan area of Sao Paulo, where approximately 23 million people live. Follow-up for at least 28 days or until hospital discharge or transfer was complete for all patients. Ninety-five (6%) patients were transferred to other hospitals before 28 days of follow-up, and at the end of data collection, on July 28, only 59 (3.9%) patients remained in the hospital.

Table 1 shows demographic characteristics and comorbidities for all patients. Symptoms and results of the most relevant laboratory tests performed at admission are shown in Tables S1 and S2, respectively, in Additional file 1. At ICU admission, the mean SAPS 3 was 64 ± 17, corresponding to 54% predicted hospital mortality in Latin America [31]. Additional file 1: Table S3 shows the management of patients on the first 24 h of ICU stay, and Additional file 1: Tables S4 and S5 show the association of baseline characteristics with survival.

**Table 1:** Baseline characteristics at ICU admission

	All, *n* = 1503	Survivors,* n* = 837	Nonsurvivors,* n* = 666	*p *value
Characteristic				
Age, y	60 ± 15	56 ± 15	64 ± 14	< 0.001
BMI	27 ± 7	28 ± 8	27 ± 7	0.002
SAPS 3	64 ± 17	59 ± 14	71 ± 16	< 0.001
SOFA	14 ± 4	13 ± 4	15 ± 4	< 0.001
Duration of symptoms, median (IQR),* d*	9 (6–12)	9 (7–12)	8 (6–13)	0.044
Glasgow coma scale	11 ± 5	12 ± 5	10 ± 6	< 0.001
Race^a^, n (%)				0.425
White	910 (61)	460 (60)	450 (61)	
Black	109 (7)	51 (7)	58 (8)	
Mix-ethnicity (Pardo)	413 (28)	212 (28)	201 (27)	
Asian	56 (4)	35 (5)	21 (3)	
Not informed	15 (1)	8 (1)	7 (1)	
Sex,* n* (%)				0.01
Male	895 (59)	431 (56)	464 (63)	
Female	608 (41)	335 (44)	273 (37)	
Comorbidities,* n* (%)				
Asthma	44 (3)	25 (3)	19 (3)	0.525
Cancer	148 (10)	45 (6)	103 (14)	< 0.001
Cardiovascular disease	221 (15)	100 (13)	121 (16)	0.077
Chronic kidney disease, dialytic	45 (3)	18 (2)	27 (4)	0.179
Chronic kidney disease, not dialytic	107 (7)	36 (5)	71 (10)	< 0.001
Chronic pulmonary disease	89 (6)	35 (5)	54 (7)	0.031
Diabetes	563 (38)	258 (34)	305 (41)	0.002
Hypertension	850 (57)	401 (52)	449 (61)	0.001

BMI: body mass index, kg/m^2^; IQR: interquartile range; SAPS 3: Simplified acute Physiology Score 3; SOFA: Sepsis-related Organ Failure Assessment. Data are presented as mean and standard deviation, unless otherwise stated; BMI missing for 137 (9%) patients; SAPS3, missing for 1 patient; SOFA missing for 8 patients

^a^The categories represent the Brazilian official race categories; Comparisons were made with t test, Mann–Whitney *U* tests or Chi-square test as appropriate

### Ventilator parameters

**Fig. 2:**
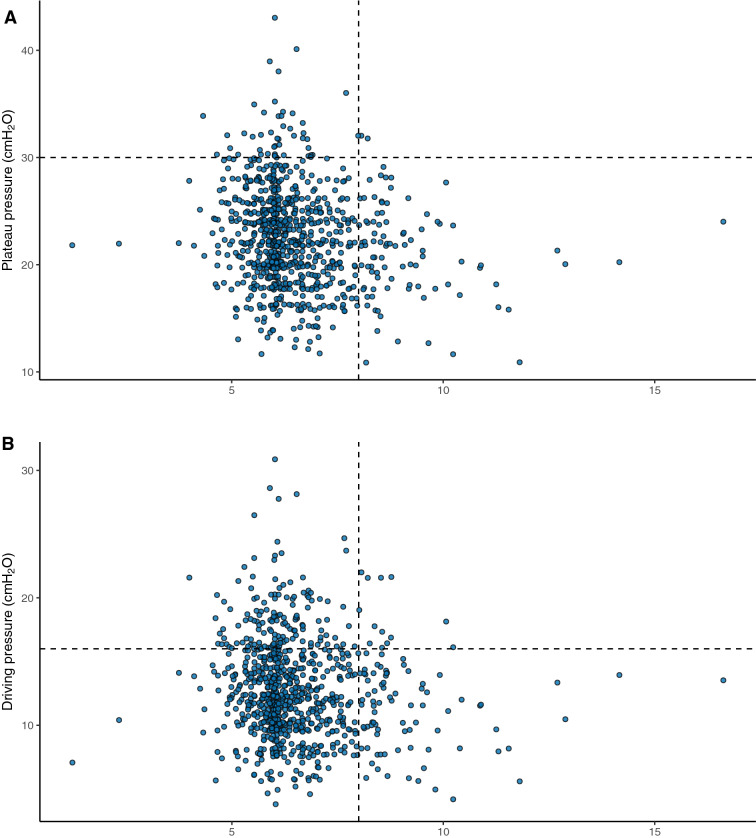

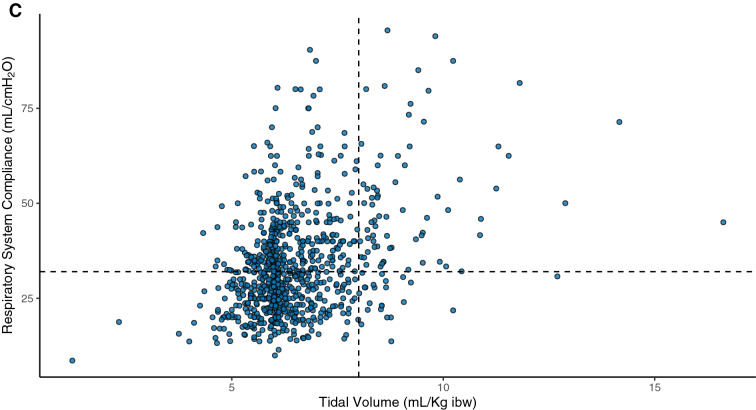
Distribution of tidal volume vs. plateau pressure (**A**), driving pressure (**B**), and compliance (**C**) for each patient on the first 24 h of ICU admission. Protective levels of ventilation, defined as tidal volume of ≤8 mL/kg of ideal body weight and plateau pressure ≤ 30 cmH_2_O, were applied to 82% of patients (lower left quadrant in panel A), and the combination of high plateau pressure (> 30 cmH_2_O) and high tidal volume (> 8 mL/kg) was rare (upper right quadrant in **A**). Using a threshold of driving pressure of < 16 cmH_2_O, 69% were ventilated within protective levels (lower left quadrant in **B**). We added subcentimetric random variability in **B** (driving pressure) to avoid overlapping of several points over the same value using the function geom_jitter, on the statistical program R

For the 984 patients under invasive mechanical ventilation in the first 24 h of ICU stay, mean tidal volume was 6.5 ± 1.3 mL/kg of ideal body weight, plateau pressure was 24 ± 5 cmH_2_O, and driving pressure was 13 ± 4 cmH_2_O (Table 2). On the first 24 h of ICU admission, 82% of patients were ventilated with tidal volume < 8 ml/Kg and plateau pressure less than 30 cmH_2_O (Fig. 2). The distribution of ventilatory parameters is shown in Additional file 1: Figure S3. Respiratory system compliance was low (Table 2), with a wide range of distribution, and did not follow a bimodal pattern (Additional file 1: Figure S3). Compliance was correlated with tidal volume, plateau pressure, and driving pressure, as shown in Additional file 1: Figures S4 and S5.

**Table 2:** Ventilatory management on the first 24 h after ICU admission, according to outcome at 28 days

Management	All, * n* = 984	Survivors,* n *= 471	Nonsurvivors,* n *= 513	*p *value
Tidal volume (mL/Kg ideal body weight), mean (SD)	6.5 ± 1.3	6.5 ± 1.3	6.5 ± 1.3	0.82
Minute volume (L/min), mean (SD)	12.0 ± 3.8	12.0 ± 4.1	12.0 ± 3.6	0.79
FIO_2_ (%), median (IQR)	50 (40–60)	45 (35–60)	50 (40–65)	< 0.001
PEEP (cmH_2_O), median (IQR)	10 (8–12)	10 (8–12)	10 (8–12)	0.647
Plateau pressure (cmH_2_O), mean (SD)	22 ± 5	22 ± 5	23 ± 5	0.033
Driving pressure (cmH_2_O), mean (SD)	13 ± 4	12 ± 4	13 ± 4	0.017
Compliance (mLcmH_2_O^−1^), median (IQR)	31.9 (24.4–40.9)	32.9 (25.0–42.0)	31.7 (23.4–40.0)	0.063
Compliance (mLcmH_2_O^−1^.Kg^−1^ ibw), median (IQR)	0.51 (0.41–0.65)	0.54 (0.42–0.67)	0.50 (0.40–0.64)	0.027
PaO_2_/FIO_2_ (%), mean (SD)	171 ± 74	178 ± 73	165 ± 75	0.004
Arterial pH, mean (SD)	7.35 ± 0.09	7.37 ± 0.09	7.33 ± 0.10	< 0.001
Arterial PaCO_2_ (mmHg), mean (SD)	44 ± 10	44 ± 9	45 ± 11	0.07
Arterial oxygen saturation (%), mean (SD)	93 ± 5	93 ± 4	92 ± 6	0.002
Rescue therapy for respiratory failure				
Prone position	154 (16)	82 (17)	72 (14)	0.577
Recruitment maneuvers	15 (2)	8 (2)	7 (1)	1.000
PEEP titration	111 (11)	61 (13)	50 (10)	0.950
Inhaled nitric oxide	2 (0)	2 (0)	0 (0)	0.582
Extracorporeal membrane oxygenation	4 (0)	1 (0)	3 (1)	0.678

SD: standard deviation; IQR: interquartile range; O_2_: oxygen; FIO_2_: inspired fraction of oxygen; PEEP: positive end-expiratory pressure; ibw: ideal body weight; PaO_2_/FIO_2_: arterial partial pressure of oxygen divided by the inspired fraction of oxygen. Data are n. (%), unless otherwise stated. PaO_2_/FIO_2_ was missing for 5 patients; Plateau pressure (cmH_2_O) and Driving pressure (cmH_2_O) were missing for 109 patients; PaCO_2_: arterial partial pressure of carbon dioxide was missing for 59 patients; arterial pH and arterial oxygen saturation were missing for 59 patients. Comparisons were made with t test, Mann–Whitney U tests or Chi-square test as appropriate

**Fig. 3:**
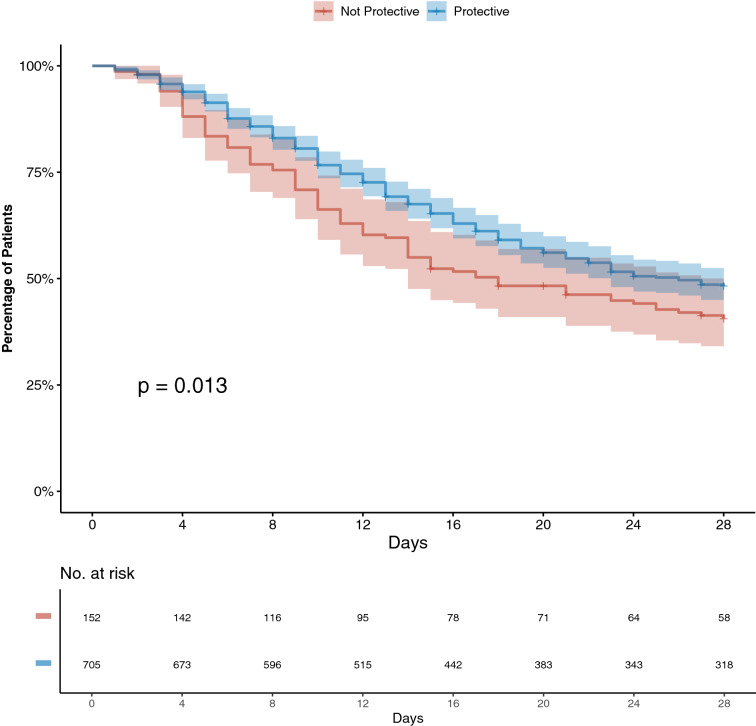
ICU survival at 28 days according to the application of protective ventilation. Solid red line represents survival of patients who received protective ventilation on day 1, defined as tidal volume of less than or equal to 8 mL/kg of ideal body weight and plateau pressure less than or equal to 30 cmH_2_O; solid blue line represents the survival of patients who were ventilated with non-protective ventilation; the shaded area represents the 95% confidence intervals. The *p *value was obtained with a Cox proportional hazards model, adjusted for PF ratio, respiratory system compliance, pH, and PEEP

Several ventilatory variables were associated with mortality, including plateau pressure, driving pressure, PF ratio, pH, and compliance (Table 2). Protective ventilation was associated with survival with a crude hazard ratio (HR) = 0.763 (95%CI 0.605–0.963). In the multivariable analysis based on a conceptual model including relevant respiratory covariates that are potential confounders, protective ventilation remained associated with increased survival, with aHR = 0.73 (95%CI 0.57–0.94), after adjustment for PEEP, compliance, PF ratio, and pH (Fig. 3 and Additional file 1: Table S6).

A sensitivity analysis, excluding highly suspected but non-confirmed cases of COVID-19 (*n* = 126), showed similar results. An additional sensitivity analysis including base excess and use of vasoactive drugs as surrogates for shock also confirmed the association between protective ventilation and survival (Additional file 1: Table S7).

### ICU outcomes

The median ICU stay was 10 (IQR 6–18) days, and median hospital stay was 16 (IQR 11–26) days (Table 3). Of the 1503 patients, 528 (35%) needed renal replacement therapy, 1095 (73%) required vasopressors, and 279 (19%) had a thromboembolic event registered as a diagnosis during their ICU stay (Table 3). At the end of 28-days follow-up, 666 (44%, 95% CI 42%–47%) patients died in the hospital. Hospital mortality at 60 days was 49% (95%CI 46%–51%).

**Table 3:** Clinical Outcomes at 28 days

Outcomes	All, * n* = 1503	Survivors,* n* = 837	Nonsurvivors,* n* = 666	*p *value
ICU length of stay, median (IQR), d	10 (6–18)	10 (5–21)	10 (6–16)	0.119
Hospital length of stay, median (IQR), d	16 (11–26)	21 (14–33)	13 (8–19)	< 0.001
Invasive mechanical ventilation	1180 (79)	552 (66)	628 (94)	< 0.001
Duration of mechanical ventilation, median (IQR), d	10 (6–17)	9 (5 – 18)	11 (6–16)	0.303
Prone positioning	427 (36)	201 (36)	226 (36)	0.570
Use of noninvasive ventilation^a^	320 (21)	165 (20)	155 (23)	0.012
Use of high-flow nasal cannula^a^	139 (9)	84 (10)	55 (8)	0.031
Extracorporeal membrane oxygenation	10 (1)	3 (1)	7 (1)	0.186
Vasopressors	1095 (73)	478 (57)	617(93)	< 0.001
Renal replacement therapy	528 (35)	187 (22)	341 (51)	< 0.001
Tracheostomy	169 (11))	116 (14)	53 (8)	< 0.001
Delirium	223 (15)	119 (14)	104 (16)	0.494
Ventilator-associated pneumonia	393(26)	175 (21)	218 (33)	< 0.001
Thromboembolic event	279 (19)	143 (17)	136 (20)	0.11
Cardiac arrhythmia	263 (17%)	101 (12)	162 (24)	< 0.001
Treatment withhold or withdraw during ICU stay	288 (19)	55 (7)	233 (35)	< 0.001

ICU: intensive care unit; IQR: interquartile range; Data are* n*. (%), unless otherwise stated

^a^To avoid intubation or prior to intubation. Comparisons were made with t test, Mann–Whitney U tests or chi-square test as appropriate

## Discussion

In this observational study including 1503 patients with COVID-19 admitted to the ICUs of the largest public hospital in Sao Paulo, we found that the 28-day mortality rate was 44% (95%CI 42–47) and 60-day mortality was 49%. Invasive mechanical ventilation was used for 79% of patients, vasopressors for 73%, and renal replacement therapy for 35%. Protective ventilation was used for 82% of patients receiving mechanical ventilation on the first 24 h of ICU stay and was independently associated with increased survival.

This is the first large cohort study of patients COVID-19 in a low- and middle-income country (LMIC) and describes the outcomes of patients treated in a large academic hospital in the context of a state preparedness plan. The hospital was the primary referral center for critically ill patients with COVID-19 and received patients from all regions of the metropolitan area of Sao Paulo, which has a total population of over 23 million people. The hospital is public, and patients were treated at no cost in accordance with the Brazilian universal health system. The preparedness plan involved cohorting COVID-19 patients in a building dedicated for the care of these patients, the creation of surge ICUs, and hiring or reallocation of healthcare professionals.

Hospital mortality at 28 days in our study was 44% (95%CI 42%–47%), and 60-day hospital mortality was 49% (95%CI 46%-51%), lower than the mortality found in a large epidemiological study based on a nationwide database with more than 250,000 cases across Brazil, which found 57% mortality for patients admitted to the ICU [34]. These figures are comparable to previous reports, showing wide variability in mortality [3–11], and reflecting differences between countries and health systems [35]. Importantly, many previous studies reported the mortality rate, while a considerable proportion of patients were still in the hospital, therefore underestimating mortality. In our study, patients were followed for at least 28 days, only 6% were transferred before 28 days, and 3.9% were still in the hospital at the end of follow-up. A high mortality rate was expected, as studies show that the burden of critical illness is higher in LMICs [36, 37] and large epidemiological studies performed in several ICUs across Brazil found high mortality for patients under mechanical ventilation [38] and for patients with sepsis [39]. In addition, our study was conducted during the first surge of cases, when no treatment was known to be effective, and mortality was higher [40]. Corticosteroids were used for only 25% of patients, since the results of the large randomized-controlled trial that showed that dexamethasone reduced mortality in hospitalized patients were released in mid-June, close to the end of our study period [41].

At ICU admission, the median duration of symptoms was 9 days, which is longer that most series [3, 10, 13], 39% of patients were already receiving vasopressors, and 60% were under invasive mechanical ventilation. Organ dysfunction on the first 24 h of admission, measured by SOFA, was higher than in most reports [3, 20, 21]. None of the previous studies reported SAPS 3, but a few studies report APACHE II of 13 to 16 [3, 20, 21], corresponding to 25% expected hospital mortality. These findings show that admission to the ICU was delayed, which may have contributed to high severity of disease at admission and higher mortality, and reflect barriers to access to health care in LMICs.

Gas exchange was severely compromised, as shown by the median PaO_2_/FIO_2_ of 171, compatible with moderate Acute Respiratory Distress Syndrome (ARDS). Ventilatory parameters on day 1 were similar to other reports [13, 20, 21], and within protective levels for 82% of patients. Adherence to a protective ventilation strategy, which is recommended by experts [42] and by the institutional protocol, was not complete but was reasonably high, and consistent with what was observed for ARDS [43] and for COVID-19 patients [21, 22]. In contrast, using more liberal tidal volumes, under the assumption that COVID-19 patients may have near-normal compliance, as recently proposed [17], has not been proven to confer protection and may have contributed to nonadherence to protective ventilation in our study.

We found associations of several ventilatory variables, including plateau pressure and driving pressure with mortality in COVID-19, similarly to what has been previously shown for ARDS due to other causes [43]. Interestingly, when ventilatory parameters were assessed one at a time, plateau pressure and driving pressure, but not tidal volume, or PEEP, were associated with mortality. This finding is compatible with our observation that limitation of both tidal volume and plateau pressure conferred an advantage not only in terms of lower tidal volume and plateau pressures but also in terms of lower driving pressures.

Compliance on day 1 was moderately low and had a wide distribution. This pattern does not support recently proposed conceptual models of two phenotypes in ARDS caused by COVID-19 [14–17]. Our findings are in line with most recently published studies in COVID-19 patients, which showed lower respiratory system compliances in COVID-19 patients [20–22]. It is possible that findings of normal compliance in severe respiratory failure in COVID-19 were influenced by the small sample sizes in early studies and timing from disease onset until the compliance measurements.

In our study, compliance was associated with mortality and provided relevant information to describe the application of protective ventilation. We found that for patients with lower compliance, non-protective ventilation was most commonly due to higher plateau pressure and driving pressure, while for patients with higher compliance, non-protective ventilation was most commonly due to higher tidal volumes. PEEP levels were moderate, in contrast to some reports of need for high PEEP [4, 10]. Prone position and PEEP titration were the most common advanced therapies used for respiratory failure on the first 24 h of ICU stay, similar to another large cohort of COVID-19 patients [21]. As per the institutional protocol, prone was indicated for all patients with PF ratio < 150 mmHg unless they had a contraindication, and was used for 36% of patients during ICU stay. The relatively low use of prone, given the severity of patients, may have impacted survival and could be related to high burden of care during the surge of cases. Similar findings were reported in large cohorts from northern Italy [4] and New York city [10], which showed that prone was used in 17% and 27% of patients, respectively.

Most patients needed advanced life support, reflected by a high incidence of use of invasive mechanical ventilation and vasopressors. Noninvasive ventilation was used for only 21% of patients prior to intubation. This finding could be due to high severity of disease at admission, lack of resources and concerns with aerosolisation with noninvasive ventilatory methods. Our findings are in line with a large multicentric study across several ICUs in Brazil, showing that noninvasive ventilatory support use increased over 8 months after the first surge of cases and was associated with decreased mortality [40]. Renal replacement therapy was used for 35% of patients, which is associated with high cost and higher burden of care, in addition to high mortality.

Our study has several important limitations: it was performed at a single-center, a large academic hospital with an institutional protocol that included ventilatory management, and therefore, the results may not be generalizable to other hospitals in Brazil. However, patients were referred from all regions of the metropolitan area, the 20 ICUs were staffed with physicians with diverse backgrounds, and some were staffed with health professionals from private hospitals in Sao Paulo who sent their teams to contribute with the state plan during the first surge of the pandemic; most patients were referred and transferred from other hospitals, possibly representing the most severe cases in Sao Paulo; part of the data were collected retrospectively, since no data were collected until we obtained study approval in our ethical committee. However, we believe that the impact on data accuracy was minimal, since we had specific electronic forms for COVID-19 symptoms in our electronic medical record, which were filled out at hospital admission for all patients, and structured ICU forms which include detailed ventilatory parameters and ICU support measures such as use of vasoactive drugs and sedation; we only collected ventilatory parameters on the first day of the mechanical ventilation, and therefore, the adherence to protective ventilation over the following days and its association with survival is unknown; we also recognize that many other practices may have impacted outcomes over the course of the study, for which we could not account; and finally, it was an observational study, and therefore, the relationship between protective ventilation and survival may be influenced by residual confounding and causality cannot be assumed. The study also has strengths: all patients admitted during the study period were included, avoiding selection bias; the sample size was large, allowing for the identification of risk factors and precise estimation of outcomes; we recorded detailed ventilatory parameters on the first 24 h of ICU stay, which allowed us to estimate the association between ventilatory strategies and survival; follow-up was long enough and complete, providing an accurate estimation of ICU survival; missing data for clinical data were minimal and quality measures provided accurate estimation of outcomes.

## Conclusions

In this single-center study performed in multiple ICUs of the largest referral hospital in Sao Paulo for COVID-19 patients during the first surge of the pandemic, patients had a high severity of disease, most needed invasive ventilation and vasopressors, and mortality was high. Protective ventilation in the first 24 h of ICU stay was associated with increased survival. Supportive care in the ICU remains the standard of care for severe cases, and COVID-19 will continue to put a high burden on health care systems around the globe, highlighting the need for a preparedness plan, development of institutional protocols that include protective mechanical ventilation and rational resource allocation.

## Supplementary Information

**Additional file 1. **Additional methods, tables, figures, references.

## Data Availability

Our institution has an institution-wide data management plan for COVID-19 datasets which includes making anonymized data publicly available to contribute to nationwide and international registries of COVID-19 patients according to a pre-defined schedule.

## Abbreviations

COVID-19: Coronavirus disease
aHR: Adjusted hazard ratio
ICU: Intensive care unit
LMIC: Low- and middle-income countries
SAPS3: Simplified Acute Physiology Score 3
SOFA: Sepsis-related Organ Failure Assessment
FIO_2_: Inspired fraction of oxygen
STROBE: Strengthening The Reporting of Observational Studies in Epidemiology
IQR: Interquartile range
BMI: Body mass index
ARDS: Acute Respiratory Distress Syndrome
