# Supplementary material for: Protective ventilation and outcomes of critically ill patients with COVID-19: a cohort study
Source: Ann Intensive Care. 2021 Jun 7;11:92. doi: 10.1186/s13613-021-00882-w (PMC8182738; doi:10.1186/s13613-021-00882-w)
Supplement: Supplementary file 1 — Additional file 1. Additional methods, tables, figures, references. [file 13613_2021_882_MOESM1_ESM.docx]

**Additional file 1**

**Protective ventilation and outcomes of critically ill patients with COVID-19: a cohort study**

**Table of contents**

1. List of investigators --------------------------------------------------------------------------1
2. Supplementary Methods -------------------------------------------------------------------3
3. Supplementary Tables ----------------------------------------------------------------------8
4. Supplementary Figures -------------------------------------------------------------------15
5. Supplementary References --------------------------------------------------------------20
6. **List of investigators**

**Writing group for the EPICCoV (EPI**demiology of **C**ritical **COV**ID-19**) study:**

Juliana C Ferreira, Yeh-Li Ho, Bruno A P Besen, Luiz MS Malbuisson, Leandro U Taniguchi, Pedro V Mendes, Eduardo L V Costa, Marcelo Park, Renato Daltro-Oliveira, Roberta M L Roepke, Joao M Silva-Jr., Maria J C Carmona, Carlos R R Carvalho.

**EPICCoV study group investigators:** Adriana Hirota, Alberto Kendy Kanasiro, Alessandra Crescenzi, Amanda Coelho Fernandes, Anna Miethke-Morais, Arthur Petrillo Bellintani, Artur Ribeiro Canasiro, Bárbara Vieira Carneiro, Beatriz Keiko Zanbon, Bernardo Pinheiro De Senna Nogueira Batista, Bianca Ruiz Nicolao, Bruno Adler Maccagnan Pinheiro Besen, Bruno Biselli, Bruno Rocha De Macedo, Caio Machado Gomes De Toledo, Carlos Roberto Ribeiro De Carvalho, Caroline Gomes Mol, Cassio Stipanich, Caue Gasparotto Bueno, Cibele Garzillo, Clarice Tanaka, Daniel Neves Forte, Daniel Joelsons, Daniele Robira, Eduardo Leite Vieira Costa, Elson Mendes Da Silva Júnior, Fabiane Aliotti Regalio, Gabriela Cardoso Segura, Giulia Sefrin Louro, Gustavo Brasil Marcelino, Yeh-Li Ho, Isabela Argollo Ferreira, Jeison de Oliveira Gois, Joao Manoel Da Silva Junior, Jose Otto Reusing Junior, Julia Fray Ribeiro, Juliana Carvalho Ferreira, Karine Vusberg Galleti, Katia Regina Silva, Larissa Padrao Isensee, Larissa dos Santos Oliveira, Leandro Utino Taniguchi, Leila Suemi Letaif, Lígia Trombetta Lima, Lucas Yongsoo Park, Lucas Chaves Netto, Luciana Cassimiro Nobrega, Luciana Haddad, Ludhmila Hajjar, Luiz Marcelo Malbouisson, Manuela Cristina Adsuara Pandolfi, Marcelo Park, Maria José Carvalho Carmona, Maria Castilho Prandini H De Andrade, Mariana Moreira Santos, Matheus Pereira Bateloche, Mayra Akimi Suiama, Mayron Faria de Oliveira, Mayson Laercio Sousa, Michelle Louvaes Garcia, Natassja Huemer, Pedro Mendes, Paulo Ricardo Gessolo Lins, Pedro Gaspar Dos Santos, Pedro Ferreira Paiva Moreira, Renata Mello Guazzelli, Renato Batista Dos Reis, Renato Daltro De Oliveira, Roberta Muriel Longo Roepke, Rodolpho Augusto De Moura Pedro, Rodrigo Kondo, Samia Zahi Rached, Sergio Roberto Silveira Da Fonseca, Thais Sousa Borges, Thalissa Ferreira, Vilson Cobello Junior, Vivian Vieira Tenório Sales, Willaby Serafim Cassa Ferreira. All investigators above are from Hospital das Clinicas HCFMUSP, Faculdade de Medicina, Universidade de Sao Paulo, SP, BR

1. **Supplementary Methods**

**Diagnosis of COVID-19**

Hospital protocol for the diagnosis of COVID-19 was based on clinical history, tomographic finding of ground glass suggestive of COVID-19 plus confirmation with RT-PCR for SARS-CoV-2. RT-PCR assays were collected from nasal and throat-swab specimens or tracheal aspirate, at admission with a minimum of 3 days of symptoms and, if negative, repeated after 48hours. As of mid-April 2020, serologic tests for IgM and IgG became available in our Institution and were performed for highly suspect cases with at least 2 RT-PCR negative samples after seven days of the onset of symptoms. If patients were admitted to the ICU as suspected COVID-19, but later had one or more negative RT-PCR and were diagnosed with other causes of respiratory failure, an infectious disease specialist reviewed the case to rule out COVID-19. When that happened, patients were transferred to other buildings in the hospital complex.

For this study, we included patients with either confirmed COVID-19, defined as a positive RT-PCR or positive serologic test (n=1377), and highly suspected COVID-19 (n=126), defined as patients with a typical history and radiological findings suggestive of COVID-19, with one or more negative RT-PCR, for whom there we no other probable diagnosis that could explain the clinical findings. Highly suspected cases in our hospital were treated as COVID-19 in isolated rooms. Because data was collected prospectively, we collected data for patients who were admitted to the ICU as suspected COVID-19, but later had COVID-19 ruled out. These patients were excluded from data analysis.

**Patient care**

Patient care was at the discretion of the ICU team but the hospital developed institutional protocols specifically for COVID-19 patients, including the use of personal protective equipment, ventilatory management, thrombosis prophylaxis and sedation.

The ventilatory protocol recommended the use of noninvasive ventilation and high-flow nasal cannula as first choice for mild cases, and intubation and invasive mechanical ventilation for patients with respiratory failure and for those who did not improve after 60 minutes of noninvasive support. The timing for intubation was at the discretion of the medical team, but so-called “early intubation” was not recommended. For patients under invasive mechanical ventilation, the protocol recommended the use of low tidal volumes (6-8mL/kg of ideal body weight) and limited plateau pressure (<30 cm H_2_O). The protocol recommended the use of a low PEEP/FIO_2_ table for PEEP titration. Prone positioning was recommended for patients with and PaO_2_/FIO_2_ <150mmHg. Recruitment maneuvers and decremental PEEP titration were suggested as rescue therapy. The ventilatory protocol was disseminated as a PDF document sent by email and instant messaging to all clinicians. The clinical protocol, together with four short tutorial videos – showing basic ventilatory management and protective ventilation, PEEP titration using the PEEP/FIO_2_ table, prone positioning, and recruitment maneuvers – were available at the hospital website in the COVID-19 resource center.

Specific drugs for treating COVID-19 were not recommended but could be used at the discretion of the attending physician. Dexamethasone was used for most patients after the publication of a clinical trial showing benefit in mid-June (E1).

**Ventilatory variables**

Ventilatory parameters were collected on day 1 and included tidal volume, respiratory rate, inspired fraction of oxygen (FIO_2_) positive end-expiratory pressure (PEEP), and plateau pressure, which was measured by the ICU team. Variables were recorded during the morning round on the first day after ICU admission (day 1). Since these parameters may vary over the course of the day, investigators were instructed to record the values applied after morning rounds discussion. For cases collected retrospectively, we recorded values registered by respiratory therapists in the electronic document filled out after morning rounds. When more than one arterial blood gas had been collected on day 1, we recorded the variables of the first blood sample of the day, and registered pH, pO_2_ and PaCO_2_. The arterial partial pressure of oxygen divided by the inspired fraction of oxygen (PaO_2_/FIO_2_) was calculated with the first blood sample of the day. Driving pressure was calculated as plateau pressure minus total PEEP. Protective ventilation was defined as ventilation with tidal volume < 8ml/Kg and plateau pressure < 30 cmH_2_O. Respiratory system compliance was obtained by dividing tidal volume, both in mLs and in mL/kg of ideal body weight, by the driving pressure. We excluded compliance values greater than 100 because these physiologically improbable values were assumed to be erroneous (n=12). Ventilatory ratio was calculated as [tidal volume × respiratory rate × PaCO_2_ / (predicted body weight × 100 × 37.5)].

**Geocoding**

We identified and geocoded (R v.3.6.2 ggmap v3.0.0) 1,586 patients’ home addresses. Specifically, patients’ zip codes available in medical charts were transformed to longitude and latitude coordinates using the Google Maps Geocoding API in the R environment. These coordinates were then plotted on a map of the city of São Paulo and the neighboring cities.

**Statistical analysis**

We tested the association of protective mechanical ventilation and other relevant ventilatory variables with survival for patients under mechanical ventilation on day 1, using Cox proportional hazards models. The multivariable model was based on a conceptual causal diagram including relevant covariates (Figure S1) (E3),

In addition, we tested the association between baseline characteristics and survival at 28 days in additional exploratory multivariate models. We performed survival analysis using the Cox proportional hazards model to identify the main risk factors for 28 days survival beyond expected associations, such as age, sex, and severity of disease at ICU admission. Variables identified a priori as possibly clinically relevant were described in the study protocol (E2) and additional variables with a P value < 0.20 in a univariate analysis were included in the multivariable Cox model.

Since this was an observational study and some laboratory tests were not performed for all patients, the association between laboratory tests at admission and survival was tested in a separate multivariable model, adjusted for age, sex and severity of disease at admission, measured by the Simplified Acute Physiology Score 3 (SAPS3). SOFA score was not added to the model to avoid collinearity, since it includes results of laboratory tests.

Finally, we performed two sensitivity analyses: in the first, we excluded the 126 patients who were highly suspected but non-confirmed cases of COVID-19 and found similar results, and in second, we added base excess and use of vasoactive drug at admission as surrogates for shock which might impact the application of protective ventilation.

The proportional hazards assumption (PHA) was tested using Schoenfeld residuals. We did not input missing data. All hypothesis tests are two-tailed with a significance level of 0.05 and performed using the R software (R Core Team, 2016, Vienna, Austria).

1. **Supplementary Tables**

**Table S1. Symptoms reported at hospital admission**

|  | **All** | **Survivors** | **Nonsurvivors** | ***p-*value** |  |
| --- | --- | --- | --- | --- | --- |
| **Symptoms, n (%)** | n=1503 | n=837 | n=666 |  | |
| Abdominal pain | 42 (3) | 24 (3) | 18 (3) | 0.972 | |
| Altered level of consciousness | 46 (3) | 20 (2) | 26 (4) | 0.123 | |
| Asthenia | 213 (14) | 131 (16) | 82 (12) | 0.077 | |
| Chills | 37 (3) | 21 (3) | 16 (2) | 1.000 | |
| Cough | 937 (62) | 531 (63) | 406 (61) | 0.351 | |
| Diarrhea | 130 (9) | 76 (9) | 54 (8) | 0.566 | |
| Fatigue | 152 (10) | 99 (12) | 53 (8) | 0.017 | |
| Fever | 780 (52) | 475 (57) | 305 (46) | <0.001 | |
| Headache | 164 (11) | 107 (13) | 57 (9) | 0.012 | |
| Loss of smell | 107 (7) | 71 (9) | 36 (5) | 0.028 | |
| Loss of taste | 71 (5) | 49 (6) | 22 (3) | 0.028 | |
| Myalgia/Arthralgia | 344 (23) | 221 (26) | 123 (19) | <0.001 | |
| Nausea/vomiting | 80 (5) | 53 (6) | 27 (4) | 0.066 | |
| Rhinorrhea | 132 (9) | 83 (10) | 49 (7) | 0.099 | |
| Shortness of breath | 1089 (73) | 612 (73) | 477 (72) | 0.557 | |
| Sore throat | 48 (3) | 35 (4) | 13 (2) | 0.022 | |

Data are counts (percentage). Comparisons were made with a chi-square test.

**Table S2 – Laboratory tests at ICU admission**

| **Laboratory test** | **All patients** | **Survivors** | **Nonsurvivors** | ***p-*value** |
| --- | --- | --- | --- | --- |
|  | **n=1503** | **n= 837** | **n= 666** |  |
| Alanine aminotransferase (U/L) | 35 (23–54) | 35 (24–54) | 34 (22–56) | 0.446 |
| Arterial lactate (mg/dL) | 15 (12–20) | 14 (11–18) | 17 (13–22) | <0.001 |
| Arterial pH | 7.38 (7.32–7.44) | 7.40 (7.34–7.45) | 7.36 (7.29–7.41) | <0.001 |
| Aspartate aminotransferase (U/L) | 44 (30–67) | 41 (29–61) | 49 (31–78) | <0.001 |
| C-reactive protein (mg/dL) | 177 (90–281) | 162 (81–271) | 187(108–295) | <0.001 |
| Creatinine (mg/dL) | 1.19 (0.78–2.32) | 0.98 (0.72–1.76) | 1.61 (0.95–3.03) | <0.001 |
| D-dimer (ng/mL) | 2147 (1096–6840) | 1763 (1014–5426) | 3318 (1280–13774) | <0.001 |
| Lymphocyte count (/mm3) | 810 (530–1190) | 920 (620–1280) | 690 (440–1040) | <0.001 |
| Platelet count (1000/mm3) | 226 (165–302) | 244 (188–324) | 202 (141–230) | <0.001 |
| Total bilirubin (mg/dL) | 0.43 (0.27–0.69) | 0.43 (0.27–0.65) | 0.44 (0.28–0.77) | 0.064 |
| White blood cells count (/mm3) | 9700 (6880–13820) | 9180 (6840–13100) | 10650 (7030–14870) | 0.001 |

Data are median (Interquartile range); *p-*value for the univariate analysis, obtained with Mann-Whitney test. Arterial lactate missing for 462 (31%) patients; Arterial pH missing for 175 (12%) patients; Aspartate aminotransferase and Alanine aminotransferase missing for 241 (16%) patients; Total bilirubin (mg/dL) missing for 268 (18%) patients; D-dimer (ng/dL) missing for 340 (25%) patients; C-reactive protein (mg/dL) missing for 235 (16%) patients.

**Table S3. Patient management on the first 24hs after ICU admission**

| **Management** | **All** | **Survivors** | **Nonsurvivors** | ***p-*value** |
| --- | --- | --- | --- | --- |
|  | n=1503 | n=837 | n=666 |  |
| Sedation |  |  |  |  |
| Midazolam | 791 (53) | 380 (45) | 411 (62) | <0.001 |
| Propofol | 173 (12) | 90 (11) | 83 (13) | 0.342 |
| Fentanyl | 816 (54) | 393 (47) | 423 (64) | <0.001 |
| Dexmedetomidine | 8 (1) | 5 (1) | 3 (1) | 0.974 |
| Vasopressors | 623 (42) | 259 (31) | 326 (49) | <0.001 |
| Respiratory support |  |  |  | <0.001 |
| Room air | 61 (4) | 49 (6) | 12 (2) |  |
| O_2_ catheter | 162 (11) | 128 (15) | 34 (5) |  |
| High flow nasal cannula | 37 (3) | 26 (3) | 11 (2) |  |
| O_2_ mask | 194(13) | 123 (15) | 71 (11) |  |
| Noninvasive mechanical ventilation | 65 (4) | 40 (5) | 25 (4) |  |
| Invasive mechanical ventilation | 984 (66) | 471 (56) | 513 (77) |  |
| Antibiotics | 1036 (69) | 564 (67) | 472 (71) | 0.163 |
| Chloroquine or hydroxychloroquine | 7 (1) | 4 (1) | 3 (1) | 1.000 |
| Corticosteroids | 373 (25) | 185 (22) | 188 (28) | 0.006 |
| Anticoagulation |  |  |  | 0.089 |
| None | 129 (9) | 60 (7) | 69 (10) |  |
| Prophylactic dose | 1225 (81) | 692 (83) | 533 (80) |  |
| Full anticoagulation | 149 (10) | 85 (10) | 64 (10) |  |

Definition of abbreviations: O_2_: oxygen; Data are n. (%). Comparisons were made with the chi-square test.

**Table S4 – Admission characteristics independently associated with 28-day survival in a multivariable Cox model**

| **Characteristic (n=1502)** | **aHR** | **Lower 95%CI** | **Upper 95%CI** | ***p*-value** |
| --- | --- | --- | --- | --- |
| Base Model | | | | |
| Age, each 10 years | 1.23 | 1.15 | 1.31 | <0.001 |
| Sex (male) | 1.21 | 1.03 | 1.42 | 0.020 |
| SAPS 3 | 1.03 | 1.02 | 1.03 | <0.001 |
| Comorbidities, organ dysfunction and symptoms | | | | |
| SOFA | 1.03 | 1.01 | 1.06 | 0.003 |
| Cancer | 1.64 | 1.30 | 2.07 | <0.001 |
| Chronic kidney disease | 1.41 | 1.12 | 1.79 | 0.003 |
| Fatigue | 0.66 | 0.50 | 0.88 | 0.004 |
| Fever | 0.79 | 0.68 | 0.93 | 0.004 |

Definition of abbreviations: aHR: adjusted hazard ratio; SAPS 3: Simplified acute Physiology Score 3; SOFA: Sepsis-related Organ Failure Assessment; *p-*values and aHRs obtained with a multivariable Cox regression model. SAPS3 was missing for 1 patient.

**Table S5 – Admission laboratory results independently associated with 28-day survival**

| **Variable (n=664)** | **aHR** | **Lower 95%CI** | **Upper 95%CI** | ***p*-value** |
| --- | --- | --- | --- | --- |
| Base model + laboratory tests | | | | |
| Arterial lactate (mg/dL) | 1.02 | 1.01 | 1.03 | < 0.001 |
| Arterial pH | 0.19 | 0.05 | 0.79 | 0.021 |
| Aspartate aminotransferase (x 10^2^ U/L) | 1.04 | 1.02 | 1.05 | <0.001 |
| White blood cells count (/mm3) | 1.05 | 1.02 | 1.07 | <0.001 |
| Lymphocyte count (x 10^3^/mm3) | 0.72 | 0.55 | 0.93 | 0.119 |
| Platelet count (x 10^4^/mm3) | 0.99 | 0.99 | 0.99 | <0.001 |

Definition of abbreviations: aHR: adjusted hazard ratio; *p-*values and aHRs obtained with multivariable Cox regression model, adjusted for age, sex and SAPS3. Arterial lactate missing for 462 (31%) patients; Arterial pH missing for 175 (12%) patients; Aspartate aminotransferase and Alanine aminotransferase missing for 241 (16%) patients; Total bilirubin (mg/dL) missing for 268 (18%) patients; D-dimer (ng/dL) missing for 340 (25%) patients; C-reactive protein (mg/dL) missing for 235 (16%) patients.

**Table S6 – Association between respiratory variables and 28-day survival in a multivariable Cox model based on the DAG conceptual model**

| **Variable (n=812)** | **aHR** | **Lower 95%CI** | **Upper 95%CI** | ***p*-value** |
| --- | --- | --- | --- | --- |
| Compliance (mLcmH_2_O^-1^) | 0.71 | 0.46 | 1.10 | 0.125 |
| PEEP (cmH_2_O) | 0.96 | 0.92 | 1.01 | 0.094 |
| pH | 0.06 | 0.02 | 0.17 | <0.001 |
| PaO_2_/FIO_2_ mmHg | 1.00 | 0.99 | 1.00 | 0.364 |
| Protective ventilation  (tidal volume < 8mL/Kg + plateau pressure <30 cmH_2_O) | 0.73 | 0.57 | 0.94 | 0.013 |

Definition of abbreviations: aHR: adjusted hazard ratio;95%CI: 95% confidence interval; PEEP: end-expiratory positive pressure; *p-*values and aHRs obtained with a multivariable Cox regression model.

There were 984 patients under mechanical ventilation on the first 24h of ICU admission. PaO_2_/FIO_2_ was missing for 5 patients; Plateau pressure and Driving pressure were missing for 101 patients; Compliance was missing for 128 patients and pH was missing for 55 patients.

**Table S7 – Association between respiratory variables and 28-day survival in a multivariable Cox model based on the DAG conceptual model+ base excess and use of vasocative drugs at ICU admission.**

| **Variable (n=784)** | **aHR** | **Lower 95%CI** | **Upper 95%CI** | ***p*-value** |
| --- | --- | --- | --- | --- |
| Compliance (mLcmH_2_O^-1^) | 0.69 | 0.44 | 1.08 | 0.102 |
| PEEP (cmH_2_O) | 0.98 | 0.94 | 1.03 | 0.428 |
| Vasoactive drug at admission | 1.31 | 1.07 | 1.60 | 0.008 |
| Base excess | 0.97 | 0.94 | 0.99 | 0.019 |
| pH | 0.30 | 0.06 | 1.43 | 0.130 |
| PaO_2_/FIO_2_ mmHg | 0.999 | 0.99 | 1.00 | 0.174 |
| Protective ventilation  (tidal volume < 8mL/Kg + plateau pressure <30 cmH_2_O) | 0.77 | 0.60 | 0.99 | 0.046 |

Definition of abbreviations: aHR: adjusted hazard ratio;95%CI: 95% confidence interval; PEEP: end-expiratory positive pressure; *p-*values and aHRs obtained with a multivariable Cox regression model.

There were 984 patients under mechanical ventilation on the first 24h of ICU admission. PaO_2_/FIO_2_ was missing for 5 patients; Plateau pressure and Driving pressure were missing for 101 patients; Compliance was missing for 128 patients; pH was missing for 55 patients and base excess was missing for 84 patients.

1. **Supplementary Figures**

**Figure S2. Causal diagram in the format of directed acyclic graph (DAG) showing the conceptual model of association between protective ventilation, other relevant covariates and survival**


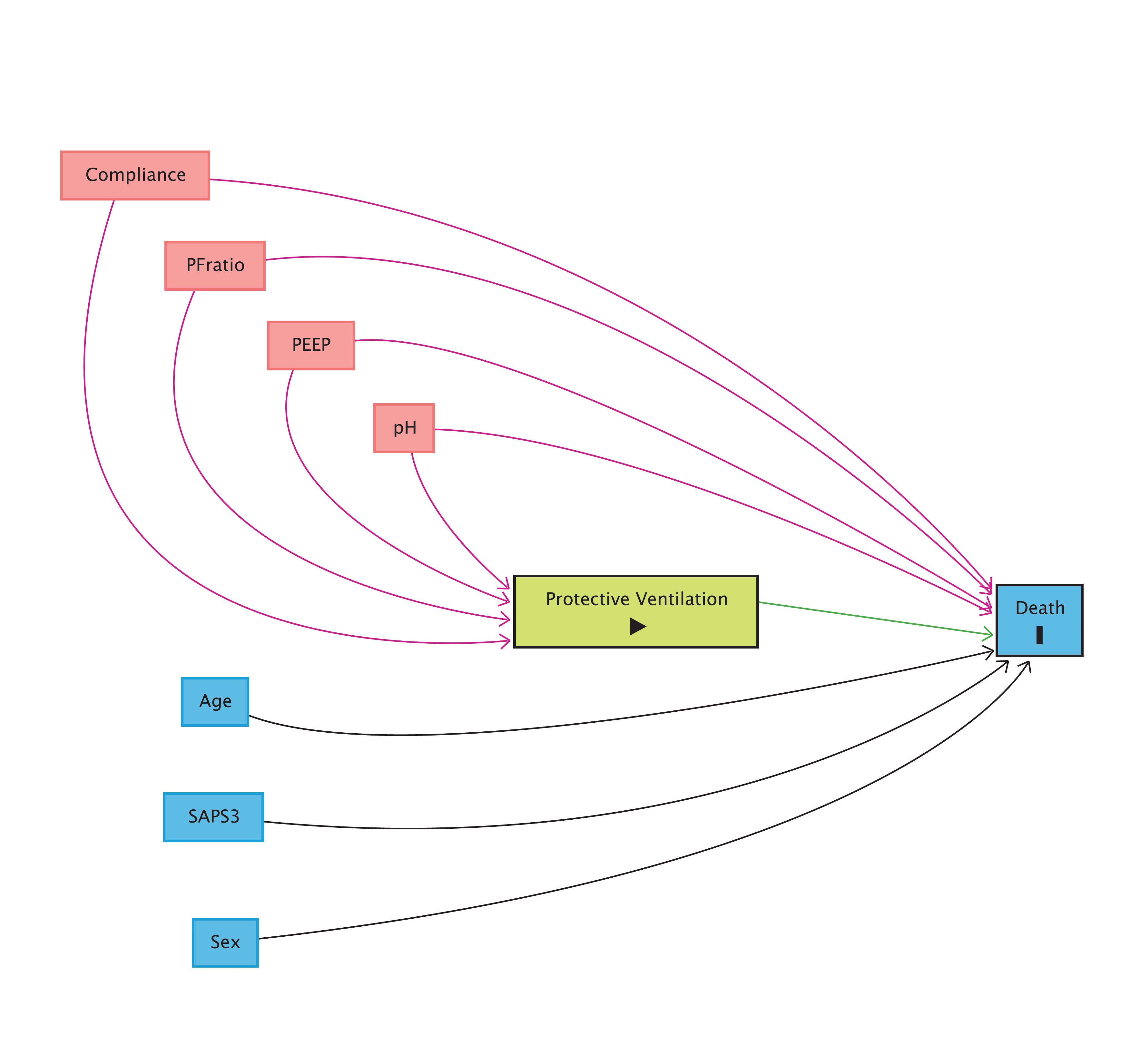


**Legend:** This conceptual model using Directed acyclic graph (DAG) shows clinically relevant variables associated with survival. Arrows indicate a suspected direct causal effect of one variable on another variable. Protective ventilation is the main predictor, shown in green; variables associated with the outcome, but not associated with the main predictor, are shown in blue; variables associated with both the outcome and the main predictor, shown in red, are potential confounders (arrows indicated a suspected direct causal effect of that variable on both the main predictor and the outcome). A multivariable analysis of the effect of protective ventilation on survival show be adjusted for all potential confounders.

**Figure S2. Map of the Sao Paulo metropolitan area and origin of transferred patients**


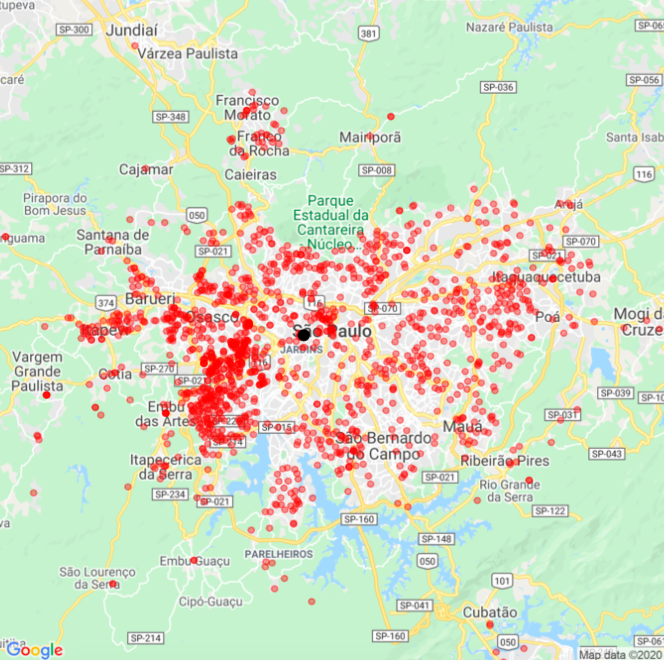


**Legend:** Map of the Sao Paulo metropolitan area, comprising 7,946km^2^ where approximately 23 million people live. Red circles represent origin of patients transferred to our hospital, showing that our hospital received patients from all districts. Intensity of color in each circle represents number of patients living in a given region. The black dot represents the location of the hospital. Origin was missing for three patients.

**Figure S3. Distribution of Respiratory Measures on the first 24 hours of ICU admission**

| **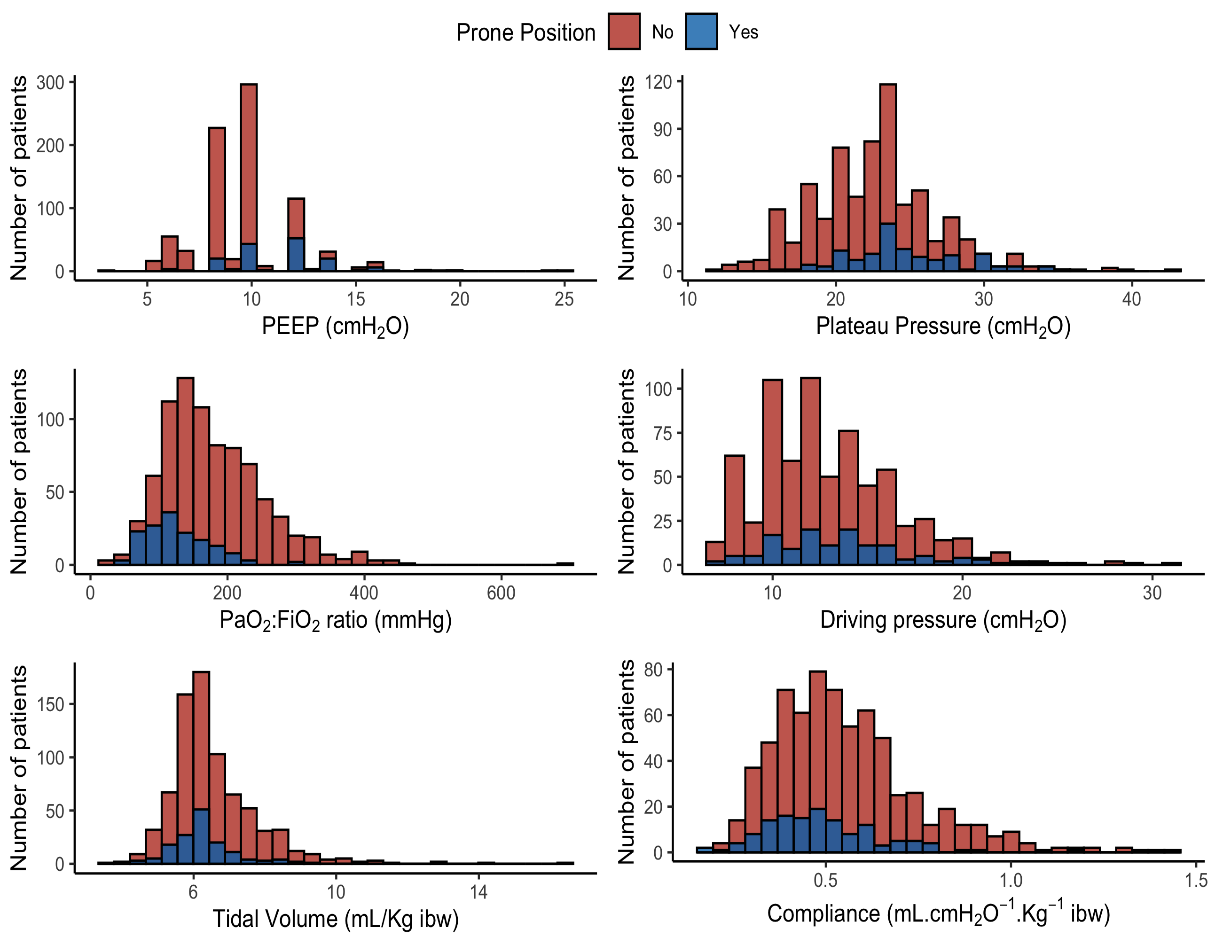** |
| --- |
| **Legend:** PEEP, positive end-expiratory pressure; FIO_2_, fraction of inspired oxygen; PaO_2_, arterial partial pressure of oxygen. There were 984 patients under mechanical ventilation on the first 24h of ICU admission. PaO_2_/FIO_2_ was missing for 5 patients, Compliance was missing for 128 patients, Plateau pressure and Driving pressure were missing for 101 patients. |

**Figure S4. Cumulative frequency distribution of tidal volume according to compliance**

| 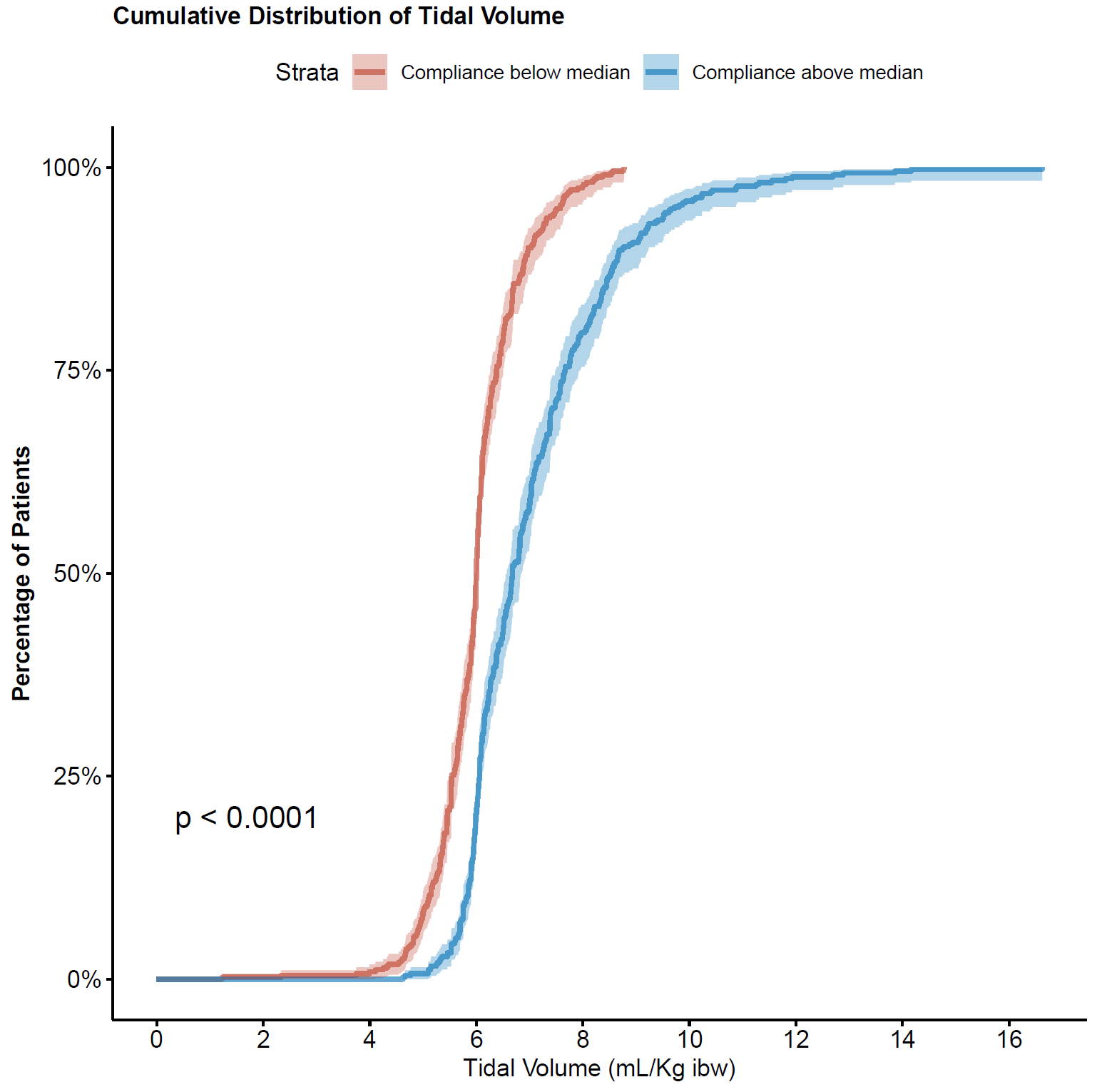 |
| --- |
| **Legend:** Cumulative frequency distribution of tidal volume according to lower (red line) or higher (blue line) respiratory system compliance. Shaded area represents 95% confidence intervals. Tidal volume was lower than 8mL/Kg of ideal body weight for 89% of patients. |

**Figure S5. Cumulative frequency distribution of driving pressure according to compliance**

| 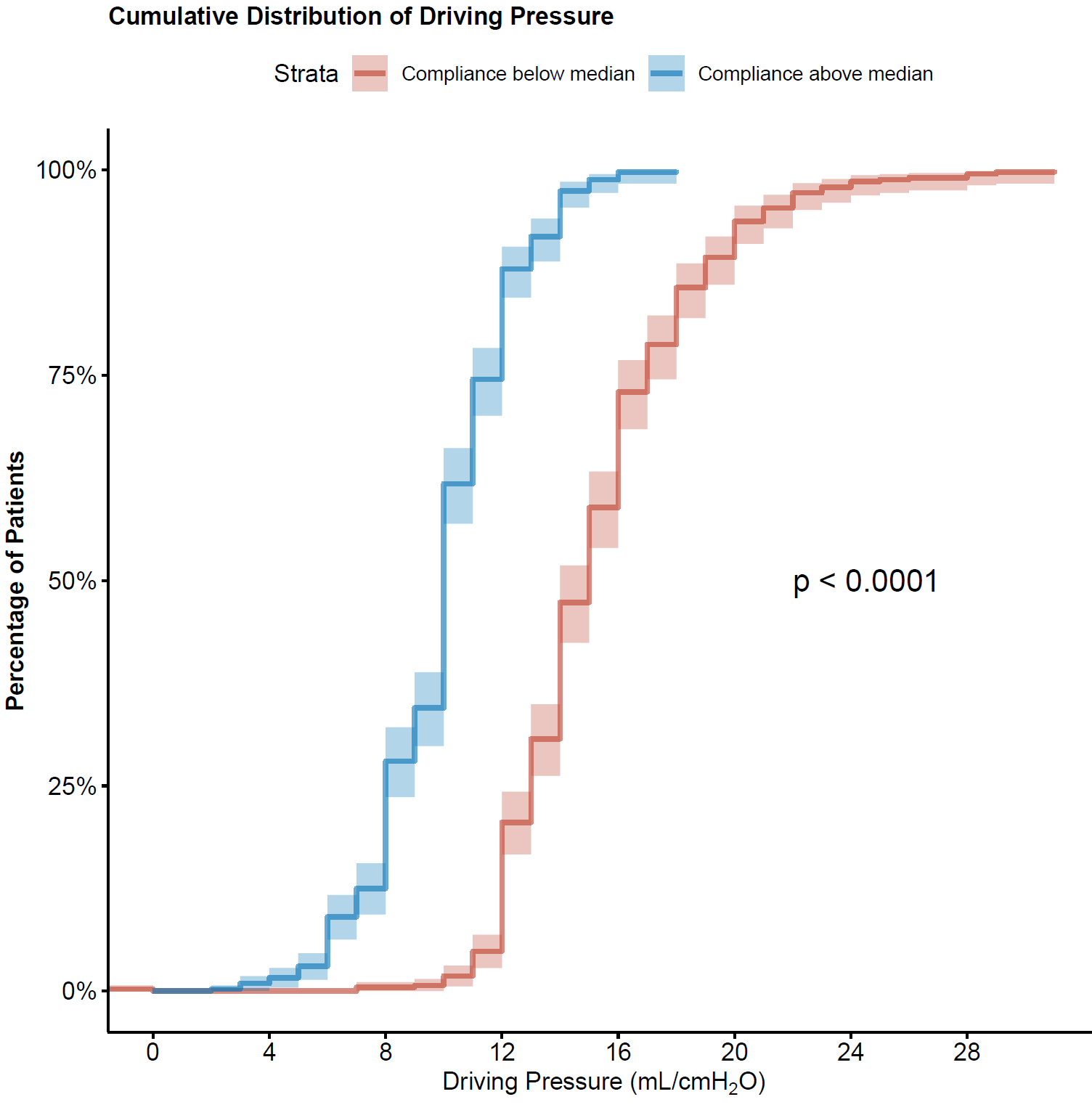 |
| --- |
| **Legend:** Cumulative frequency distribution of driving pressure according to lower (red line) or higher (blue line) respiratory system compliance. Shaded area represents 95% confidence intervals. Driving pressure was < 16 cm H_2_O for 78% of patients. |

1. **Supplementary References**

E1. Oxford University – Recovery study news release, available at: <https://www.ox.ac.uk/news/2020-06-16-dexamethasone-reduces-death-hospitalised-patients-severe-respiratory-complications>#

E2. Ferreira JC, Ho Y-L, Besen BAMP, et al. Characteristics and outcomes of patients with COVID-19 admitted to the ICU in a university hospital in São Paulo, Brazil - study protocol. Clinics 2020;75:e2294.

E3. Textor J, van der Zander B, Gilthorpe MS, Liskiewicz M, Ellison GT. Robust causal inference using directed acyclic graphs: the R package 'dagitty'. Int J Epidemiol. 2016;45(6):1887-1894.
